# The Effect of Age on Peri-Operative Outcomes after FEVAR

**DOI:** 10.3390/jcm12113858

**Published:** 2023-06-05

**Authors:** Amun Georg Hofmann, Maria Elisabeth Leinweber, Afshin Assadian, Juergen Falkensammer, Fadi Taher

**Affiliations:** 1 Department of Vascular and Endovascular Surgery, Klinik Ottakring, Montleartstrasse 37, Pavillon 30B, 1160 Vienna, Austria; 2 Department of Vascular Surgery, Barmherzige Brueder Hospital, 4020 Linz, Austria

**Keywords:** FEVAR, octogenarians, endovascular aortic repair, aortic aneurysm, juxtarenal, pararenal

## Abstract

Introduction: Fenestrated endovascular aortic repair (FEVAR) has become a popular custom-made treatment option for juxtarenal and pararenal aneurysms. It has been previously investigated whether octogenarians as a distinct subgroup are at increased risk for adverse outcomes after FEVAR. With diverging results and an inconclusive understanding of age as a risk factor in general, an analysis of the historical data of a single center was conducted to add to the available body of evidence and further investigate the effect of age as a continuous risk factor. Methods: A retrospective data analysis of a prospectively maintained single-center database of all patients who underwent FEVAR at a single department of vascular surgery was performed. The main endpoint was post-operative survival. In addition to association analyses, potential confounders such as co-morbidities, complication rates, or aneurysm diameter were examined. In terms of sensitivity analyses, logistic regression models were created for the dependent variables of interest. Results: During the observation period from April 2013 to November 2020, 40 patients over the age of 80 and 191 patients under the age of 80 were treated by FEVAR. The 30-day survival showed no significant difference between the groups (95.1% in octogenarians and 94.3% in patients under 80 years of age). The sensitivity analyses conducted also showed no difference between the two groups, and complication and technical success rates were comparable. The aneurysm diameter was 67 ± 13 mm in the study group and 61 ± 15 mm in those under 80 years of age. Additionally, the sensitivity analyses showed that age as a continuous variable exhibits no effect on the outcomes of interest. Discussion: In the present study, age was not associated with adverse peri-operative outcomes after FEVAR, including mortality, lower technical success rates, complications, or length of hospital stay. Essentially, the most highly associated factor with hospital and ICU length of stay was time spent in surgery. However, octogenarians had a significantly larger aortic diameter at the time of treatment, which might indicate the potential introduction of bias by pre-interventional patient selection. Nevertheless, the usefulness of research on octogenarians as a distinct subgroup might be questionable regarding the scalability of results, and future studies might focus on age as a continuous risk factor instead.

## 1. Introduction

In 2015, the World Health Organization published *The World Report on Ageing and Health*, highlighting the dramatic increase in the number and proportion of elderly people in societies worldwide. By 2050, the proportion of the population aged >60 years is expected to exceed 30% in several countries in Europe, North America, China, Thailand, Vietnam, Chile, and Iran [1]. The United States is estimated to have over 83.7 million people aged over 65 years by 2050, with those aged over 85 years accounting for 4.5% of the overall population [2].

As people age, the prevalence of vascular diseases, such as carotid artery stenosis, peripheral artery disease, and abdominal aortic aneurysms (AAA), rises significantly, with over 20% of octogenarians and 30% of nonagenarians exhibiting vascular pathologies in at least one arterial segment [3]. According to data from 51,153 AAA patients collected from 11 different countries by the International Consortium of Vascular Registries, octogenarians accounted for over 23% of all treated AAA patients between 2010–2013 [4]. The increasing proportion of elderly patients with reduced physiological reserve and resistance requiring vascular care has led to growing concern regarding treatment strategies for this population [5].

Developments over the past two decades have improved the treatment of patients with AAA in many ways. The former reference standard of open aortic surgery has been supplemented by alternative endovascular aortic repair (EVAR). EVAR has expanded the treatment options for multimorbid and frail vascular patients who may not have been considered for elective AAA repair before [6]. For instance, an analysis of the US Nationwide Inpatient Sample showed a significant increase in elective AAA repairs in patients aged ≥85 years, which was attributed mainly to a general increase in the use of EVAR in clinical practice [7].

EVAR has been associated with lower morbidity and peri-operative mortality than open aortic repair and may bring other advantages, such as shorter operating time and duration of in-hospital stay. A meta-analysis from 2017 comprising 25,723 AAA patients also showed acceptable peri-operative and midterm mortality rates with similar rates of technical success and 5-year re-interventions in octogenarians undergoing EVAR, indicating endovascular aneurysm repair to be a suitable and safe treatment strategy in this population [8].

However, anatomical prerequisites exist in order to be able to perform EVAR within instructions for use with “off-the-shelf” prostheses. There is some published evidence that non-compliance with the required criteria might also be associated with poor technical or clinical results [9,10]. This may apply in particular to the proximal sealing while preserving perfusion to outgoing branches of the aorta and end-organ function.

Fenestrated endovascular aortic repair (FEVAR) using a custom-made graft provides an endovascular treatment option for juxtarenal or pararenal aortic pathologies. Based on thin-slice computed tomography angiography (CTA) images of affected patients, a prosthesis with precisely placed fenestrations can be manufactured so that the landing zone of the stent prosthesis is no longer limited to the infrarenal aorta. In juxtarenal or pararenal aneurysms without an infrarenal anchoring zone, the landing zone can be relocated using this technique to a more suitable anatomical location, namely the suprarenal, paravisceral, or supratruncal aorta, to achieve a sufficient proximal seal. The exact placements of the fenestrations are important, and the test implantation procedure of a non-sterile prototype of the planned prosthesis in a three-dimensional model of the patient’s aorta can help to achieve a high level of technical success [11]. More modern types of testing also include numerical simulations with finite-element analyses that could be equivalent but also have the potential to save time in prosthesis manufacturing and simplify communication between the treating surgeon and the engineers who oversee prosthesis planning and production [12]. There are also anatomical criteria for FEVAR that must be met in order to use the technique within its instructions for use and achieve long-lasting aneurysm exclusion. It has previously been shown that many patients treated using this technique cannot necessarily be treated using an alternative chimney technique (without a custom-made prosthesis and with parallel stent grafts) [13].

FEVAR, as an option to further pursue an endovascular treatment strategy using customized grafts when off-the-shelf prostheses are not feasible, has undergone rapid popularization over the last decade. Previous studies have generated encouraging clinical data on the feasibility and safety of FEVAR for a variety of aortic pathologies [14,15,16,17,18]. The current study aims to investigate the results of the procedure in octogenarians at a single department of vascular and endovascular surgery. While some previously published data suggest the procedure to be safe and feasible in elderly patients, there have, however, been reports of worse outcomes in octogenarians [19,20,21]. The current retrospective analysis aims to contribute to the growing body of literature on FEVAR results by adding additional clinical experience on patient characteristics and data on technical success, complication rates, and survival of octogenarians after FEVAR. The study aims to report the ratio of octogenarians among patients treated by FEVAR at a single center, to identify differences in aneurysm morphology among this group and patients under the age of 80 years undergoing FEVAR, and to identify possible differences in length of intensive care unit and hospital stay, as well as technical and clinical outcome.

## 2. Materials and Methods

### 2.1. IRB Approval

The study was approved by the appropriate institutional review board and ethics committee of the city government of Vienna. The approval included a waiver of informed consent for this retrospective analysis. The study was conducted according to the Declaration of Helsinki.

### 2.2. Design

The present investigation is a retrospective single-center cohort study.

### 2.3. Participants

All consecutive patients undergoing FEVAR at a single department of vascular and endovascular surgery during the study period from April 2013 to November 2020 were retrospectively included in the analyses. Due to the excess mortality rates observed in Austria during the COVID pandemic, starting with the second local wave (December 2020), we only included patients until then to minimize confounding. Patients aged 80 years or older at the time of surgery comprise the study group of octogenarians, while patients under the age of 80 act as the non-octogenarian control group.

### 2.4. Analysis

Patient characteristics were analyzed by descriptive statistical methods, including the calculation of measures of central tendency and dispersion. Association analysis was performed by calculating odds ratios (OR) with 95% confidence intervals or Pearson’s correlation coefficient. Trends were assessed by chi-square test for trend, and *t*-tests were used to compare group means. Regression analyses included univariate linear regressions as well as multivariate logistic regression. We defined statistically significant results as *p*-value < 0.05 and 95%Cis, not including 1.0. All statistical analyses were performed with R version 4.1.3 (R Foundation for Statistical Computing, Vienna, Austria) in RStudio (Posit PBC, Boston, MA, USA).

## 3. Results

### 3.1. Sample Characteristics

**Figure 1:**
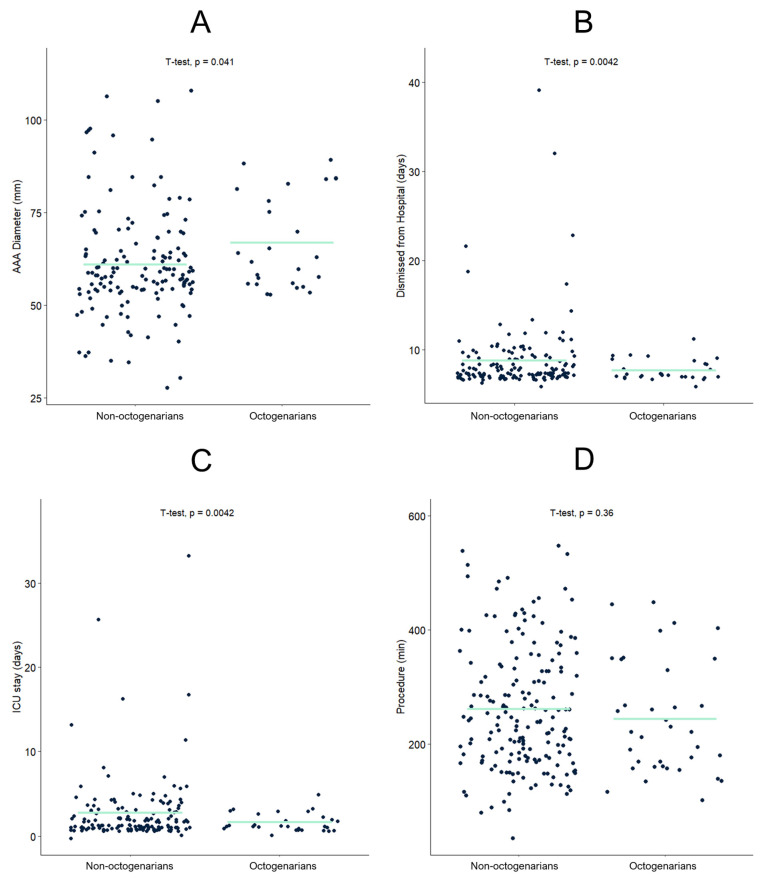
Comparative analysis of octogenarians and non-octogenarians regarding maximum aneurysm diameter (**A**), days spent in the hospital after surgery (**B**), days spent in intensive care after surgery (**C**), as well as time in surgery (primary procedure) (**D**) (AAA = abdominal aortic aneurysm). Turquoise line indicates group means.

In total, 231 patients were included in the study, with 191 below 80 years of age and 40 octogenarians. The cohorts were comparable regarding most clinical characteristics. Patient characteristics are displayed in Table 1. However, octogenarians had higher average maximum AAA diameters (mean difference 6 mm) (see Figure 1A) and shorter hospital and ICU stays after surgery (see Figure 1B,C). No statistically significant difference was found regarding time spent in surgery between groups (see Figure 1D). Patients with diameters <50 mm received treatment either due to rapid growth or because of a penetrating aortic ulcer.

### 3.2. Association of Post-Operative Mortality and Age

Thirty-day mortality was 5.2% in non-octogenarians and 5.0% in octogenarians (Table 2). No statistically relevant association between age group and 30-day survival was found (OR, 95%CI: 0.91, 0.13–3.64). To further investigate the potential association of age and post-operative survival after FEVAR, a sensitivity analysis was conducted. Multivariate logistic regression was established using age at surgery in years, AAA diameter, ASA risk stratification, and time in surgery as predictors. None of the variables suggested a significant predictive value. Neither age group at a cut-off of 80 years nor age as a continuous variable appears to be associated with post-operative survival after FEVAR in the present study population.

### 3.3. Age and Further Procedure-Related Outcomes

A comparative analysis of primary technical success rates between age groups showed no difference of statistical or clinical significance. In non-octogenarians, 28 (15.0%) cases were classified as a primary technical failure as opposed to 7 (18.9%) cases in octogenarians (OR, 95%CI: 1.33, 0.53–3.31) (7 patients missing from the sample). Similarly, there was no evidence for a difference in intra-operative complications, with observed complications (including minor) in 17.9% of cases for both groups (OR, 95%CI: 1.02, 0.38–2.42). While univariate logistic regression concerned with the effect of age on primary technical failure resulted in a significant negative association, this relationship was nullified after the inclusion of AAA maximum diameter, time spent in surgery, and ASA classification as additional covariates. The analogous workflow for intra-operative complications also showed no effect of age. Time spent in surgery was the only predictor with a significant association regarding intra-operative complications, which is intuitive considering their causative relationship.

In the present dataset, 68 patients in the non-octogenarian group (35.6%) and 13 patients in the octogenarian group (32.5%) underwent treatment with chronic kidney disease and associated impaired kidney function. Post-interventional acute kidney injury was found in 13.6% of non-octogenarians and 10.0% of octogenarians (OR, 95%CI: 0.71, 0.23–2.12) (see Table 3), indicating that octogenarians had no increased risk for post-procedural acute kidney injury.

Post-interventional morbidity rates also showed no association with age groups, as 41 (21.5%) non-octogenarians had systemic health-related events such as pneumonia or MI within 30 days after surgery compared to 7 (17.1%) octogenarians (OR, 95%CI: 0.78, 0.32–1.88).

Endoleak type I or III at first postoperative imaging and re-intervention rates (including any type of endovascular or open surgical reintervention or operation) within 30 days of the index procedure were comparable between octogenarians and non-octogenarians (12.5 vs. 8.4%, *p* = 0.3; and 10.0 vs. 16.8%, *p* = 0.2, respectively). These data regarding endoleak and reintervention rates solidify the findings that octogenarians are not associated with impaired peri-interventional outcomes after FEVAR compared to non-octogenarians.

### 3.4. Factors Associated with Hospital and ICU Stay Length

**Figure 2:**
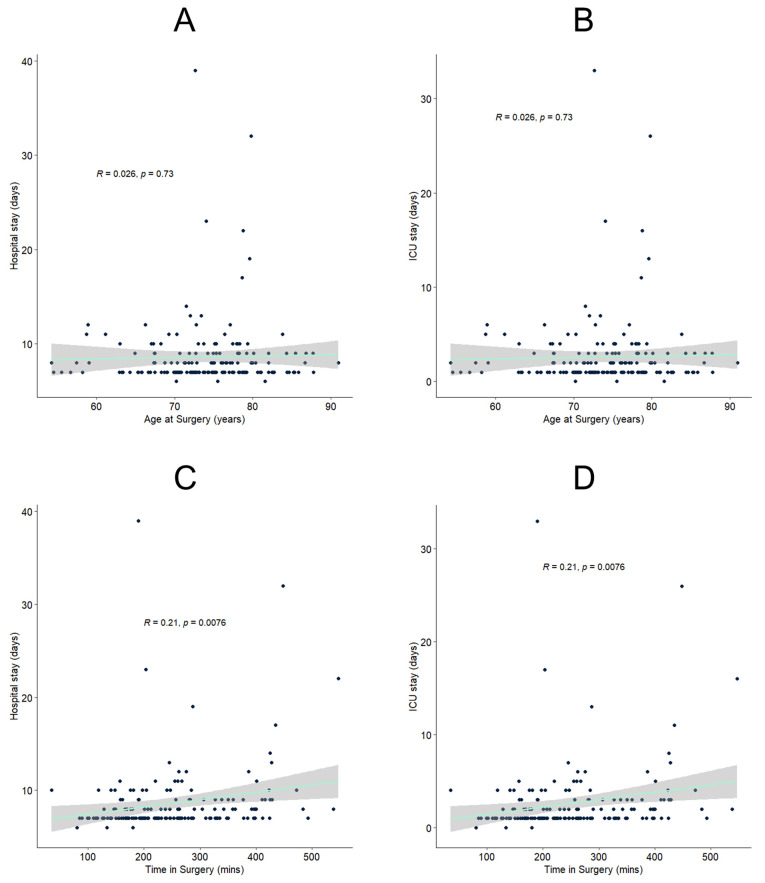
Conducted association analyses of age and hospital (**A**) as well as intensive care unit (ICU) stay (**B**), and time in surgery and hospital (**C**) as well as ICU length of stay (**D**). The turquoise line depicts a linear regression, and the shaded grey area is the corresponding 95% CI. R equals the Pearson correlation coefficient with the associated *p*-value.

The observed difference in hospital and ICU stays between octogenarians and non-octogenarians induced subsequent analyses. However, even though group-level differences regarding hospital and ICU stays were present, there was no linear association between age and both metrics (see Figure 2A,B). To investigate potential confounders, we established another multivariate logistic regression using age at surgery in years, AAA diameter, ASA risk stratification, and time in surgery as predictors. Time in surgery was found to be the only meaningful predictor. However, association analyses of procedure time and hospital as well as ICU stay only showed a low positive correlation (r = 0.21 in both cases) (see Figure 2C,D).

## 4. Discussion

In the present sample of 231 patients undergoing FEVAR, 17.3% were 80 years of age or older. They were similar regarding most clinical characteristics compared to non-octogenarians, while on average receiving treatment at larger aneurysm diameters. There was no evidence of an association between age and adverse outcomes, including peri-operative mortality, post-interventional morbidity rates including acute kidney injury, higher technical failure rates, increased complications including endoleaks and re-intervention rates, or length of hospital or ICU stay. However, hospital and ICU stays were found to have a weak correlation with time spent in surgery.

Earlier discharge from intensive care and the hospital in octogenarians may also be a result of rigorous patient selection, which may perhaps be performed more strictly in elderly patients, potentially introducing bias in the conducted analysis. However, ASA classifications and co-morbidities indicate similarities between the investigated groups regarding their overall health. Nevertheless, it is possible that anatomic features between groups act as a confounder, i.e., interventional treatment in case of unfavorable or hostile anatomy might be more likely offered to younger patients than older ones. This could at least partially explain longer surgery times and the subsequent hospital/ICU stays observed in the sample. Further limitations of the current investigation are its retrospective nature and single-center design.

Larger aneurysms in octogenarians may be a result of a later time of diagnosis but may also reflect possible differences in the decision-making process when encountered with an octogenarian with a small aneurysm as opposed to a younger patient. This cannot be investigated based on the currently available data for this report.

As the global population is aging, it is expected that by 2050 approximately 22% will be 60 years or older [22]. The need for finding solutions for an aging population is not limited to but certainly of high interest in healthcare. Age as a limitation for operative procedures has been of growing interest in clinical medicine, for example, in orthopedics for total joint replacement surgery [23,24,25]. There has been a plethora of studies suggesting an increased risk for peri-operative complications for elderly patients in several fields, ranging from cerebral aneurysms [26,27] to common abdominal procedures [28]. Apart from adverse outcomes, surgical treatment in older patients was also shown to be more resource intensive with longer hospital stays [29]. However, contrary reports that could not show a relationship between age and peri-operative adverse outcomes exist [30], and it is not conclusively understood whether the often-proclaimed association of age and adverse events is routinely affected by confounding co-morbidities and selection bias. In the case of FEVAR, a growing body of literature investigates the feasibility and safety of the procedure in octogenarians with varying results [19,20,21,31,32,33,34]. Studies largely agree that it remains a valuable option to consider in patients fit for the procedure [19,31,32], even though selected studies report an increased mortality or risk of non-home discharge in octogenarians [19,21]. However, especially in direct comparison to open aortic surgery, FEVAR is superior regarding most clinical outcomes in octogenarians [35].

What remains to be discussed in the literature is the fact that the separation of groups based on age at 80 years is essentially an arbitrary cut-off. There is no physiological or epidemiological evidence available to us suggesting that age 80 is a significant barrier to poor clinical outcomes. It appears as if octogenarians historically seemed to be used as a synonym for elderly, which as little as two decades ago was of practical nature. Nevertheless, aging populations accompanied by improved healthcare lead to a situation where octogenarians are neither rare nor necessarily in bad physical condition, questioning the usefulness of studying the mere fact of belonging to this age group as a risk factor. If age is to be investigated, it appears more reasonable to consider it a continuous variable, as we did in the course of our sensitivity analyses. This also has the benefit that could display whether any potential effect of age is linear or exponential, which in turn would lead to scalable results that could “age” better in light of even older populations in the future. Otherwise, we run at risk of repeating our research efforts two decades from now, focusing on outcomes in nonagenarians.

Our findings correspond with previously published data indicating that FEVAR is not associated with increased post-operative mortality in octogenarians. Furthermore, age does not seem to be associated with other adverse peri-operative outcomes, such as technical success rates or hospital length of stay. Our results add to the growing body of evidence that age should not be considered a limiting factor when deciding on the interventional treatment of complex aortic aneurysms from a medical perspective.

## Figures and Tables

**Table 1:** Baseline characteristics of the study population. Data are shown as mean (standard deviation) or count (percentage). Percentage for N relates to the whole sample, in the remaining rows to the respective group (ASA = American Society for Anesthesiology risk classification, CHD = coronary heart disease, ICU = intensive care unit). (*p*-value resulting from *t*-test or chi-square in case of ASA and fenestrations).

	Non-Octogenarians	Octogenarians	*p*-Value
N	191 (82.7%)	40 (17.3%)	-
Age	72.1 (5.8)	83.3 (2.7)	-
Aneurysm diameter Missing	61 (15)	67 (13)	0.041
	48 (25.1%)	14 (35.0%)	
ASA			0.040
1	1 (0.5%)	0 (0%)	
2	47 (24.6%)	2 (5.0%)	
3	132 (69.1%)	33 (82.5%)	
4	7 (3.7%)	3 (7.5%)	
Hypertension	92.2%	94.6%	0.868
Diabetes	31.2%	12.9%	0.065
CHD	48.9%	54.5%	0.792
Time in surgery	261 (107)	244 (100)	0.36
Days in ICU	2.8 (4.1)	1.7 (1.1)	0.004
Days in hospital	8.8 (4.1)	7.7 (1.1)	0.004
Fenestrations			0.426
1	11 (5.8%)	6 (15.0%)	
2	30 (15.7%)	5 (12.5%)	
3	30 (15.7%)	7 (17.5%)	
4	106 (55.5%)	20 (50.0%)	
5	13 (6.8%)	1 (2.5%)	

**Table 2:** Unadjusted association of 30-day survival between age groups.

30 Day Survival	+	−	Total
Non-octogenerians	180 (94.8%)	11 (5.2%)	191 (100%)
Octogenerians	38 (95.0%)	2 (5.0%)	40 (100%)
Unadjusted odds ratio (95% CI): 0.91 (0.13–3.64)			

**Table 3:** Post-interventional acute kidney injury.

Acute Kidney Injury	Non-Octogenarians	Octogenarians
Yes	26 (13.6%)	4 (10.0%)
No	165 (86.4%)	36 (90.0%)

## Data Availability

Data can be made available upon reasonable request to the corresponding author.
